# Soil transmitted helminths and scabies in Zanzibar, Tanzania following mass drug administration for lymphatic filariasis - a rapid assessment methodology to assess impact

**DOI:** 10.1186/1756-3305-5-299

**Published:** 2012-12-21

**Authors:** Khalfan A Mohammed, Rinki M Deb, Michelle C Stanton, David H Molyneux

**Affiliations:** 1 NTD Control Programme Manager, Ministry of Health, Zanzibar, United Republic of Tanzania; 2 Centre for Neglected Tropical Diseases, Liverpool School of Tropical Medicine, Liverpool, L3 5QA, UK

## Abstract

**Background:**

Ivermectin and albendazole are used in annual mass drug administration (MDA) for the lymphatic filariasis elimination programmes in African countries co-endemic for onchocerciasis, but have additional impact on soil transmitted helminths and the ectoparasitic mite which causes scabies. Assessing these collateral impacts at scale is difficult due to the insensitivity of available parasite detection techniques.

**Methods:**

The numbers of cases diagnosed with intestinal helminths and scabies and who received prescriptions for treatment were evaluated in 50 health centres in Zanzibar. Records were examined from 2000, prior to the initiation of MDA to 2005, after six rounds of MDA for lymphatic filariasis had taken place.

**Results:**

Health centre records showed a consistent decline in the number of cases of intestinal helminths and scabies diagnosed by community health workers in Zanzibar and the number of prescriptions issued across five age groups. A 90-98% decline in soil transmitted helminths and 68-98% decline in scabies infections were recorded. Poisson regression models aggregated to both the island-level and district-level indicated that the decline was statistically significant.

**Conclusions:**

The described method of examining health centre records has the potential for use on a large scale, despite limitations, as a rapid method to evaluate the impacts resulting from both lymphatic filariasis and onchocerciasis MDA. This would result in a reduction in the need for parasitological evaluations to determine prevalence and intensity.

## Background

In 1997, the World Health Assembly resolution (WHA50.29) called on Member States to eliminate LF as a public health problem by the year 2020 [1] and in 2000 the Global Programme to Eliminate Lymphatic Filariasis was initiated [2,3]. In Zanzibar, the Programme for Eliminating Lymphatic Filariasis (PELF) administered donated drugs, namely ivermectin (Mectizan) and albendazole, annually to all eligible individuals for six consecutive years (2001–05), through a community-based delivery system [4]. Following six rounds of MDA, in which social and religious networks were used to achieve high coverage, a marked reduction in both the microfilarial (mf) prevalence and intensity was seen in rural and urban sentinel sites from Unguja, hence showing the drug delivery strategy to be effective [4,5].

The eligibility criteria for individuals to receive treatment during MDA are that individuals are over five years of age (as determined by dose pole measured height of 90 cms as a surrogate for age 5) and in good health. Women who have recently given birth, pregnant women, the elderly and extremely ill are not eligible to receive medication.

MDA is effective at reducing prevalence and microfilarial load of *Wuchereria bancrofti* infection [2,3,5,6], but additional benefits accrue because of the impact of albendazole and ivermectin on other helminths and ectoparasites, specifically *Strongyloides stercoralis* and *Ascaris lumbricoides* (roundworm), hookworm and to a lesser extent on *Trichuris trichiura* (whipworm) (collectively the soil transmitted helminths, [STH]), and scabies respectively [7,8]. Studies by Knopp et al. [9] successfully demonstrated a decrease in prevalence of more than 95% of STHs following the delivery of benzimidazoles (albendazole and mebendazole) alone within the school health strategy system in Unguja island, Zanzibar but not on Pemba [9].

Ivermectin monotherapy reduces scabies prevalence after a single treatment, with almost complete disappearance of infection after 2 or more treatments [10]. For example, in the Solomon Islands the prevalence of scabies fell from a mean of 25% to 1% and scabies associated sores fell from 40% to 21% [7] following the administration of ivermectin. Ivermectin is used in both onchocerciasis and LF programmes in Africa [2].

The aim of this study was to test a new methodology for assessing the impact of MDA for lymphatic filariasis on the reduction in the number of cases of STH and scabies retrospectively by checking health centre records where MDA distribution had taken place. The approach adopted can be regarded as a “rapid assessment” approach. In resource poor settings it is not possible to routinely undertake large numbers of stool and skin examinations due to both cost and limited human resources. Faecal examination, the “gold standard” for parasitological diagnosis, is a relatively insensitive method, and given the impact of MDA for LF on the intensity of STH infections, that inherent lack of sensitivity will be compounded following several rounds of MDA. The use of rapid approaches as opposed to gold standard diagnoses, which are often invasive, is a diagnostic approach widely used to determine geographic distribution as well as impact of MDA on neglected diseases targeted for preventive chemotherapy [11,12].

## Methods

To determine the impact of MDA for LF on STH and scabies, 50 primary health care units (PHCU) in the ten districts of Zanzibar were chosen. Five PHCUs were selected at random in each district and the record registers were used to obtain data of registered cases of STH and scabies during a six year period (2000–2005). Thirty PHCUs were on Unguja Island and 20 on Pemba. The information collected in 2000 formed the baseline data for the number of cases identified prior to the initiation of the MDA programme.

Permission to access these records was obtained from the Ministry of Health and Social Welfare, Zanzibar. Data acquired from patient registers included the name, age, sex, complaint and treatment of individuals who visited the PHCU. The register for each year and health facility was systematically examined by two data extractors to reduce the potential for human error resulting from reading handwritten patient registers completed by health staff. The data extractors were also provided with specific guidelines on what could be considered reliable data. Drugs had been provided to all individuals attending PHCUs, either at the health facility itself or from the local pharmacies if a prescription was required. Stool examinations were not conducted due to the limited sensitivity of the Kato-Katz method for determining prevalence and intensity particularly after 6 years of MDA with high coverage [5].

### Case-level analysis

Data from five age groups was obtained: birth-5 years, 6–10 years, 11–15 years, 16–20 years and over 20 year olds. Children in the birth-5 years age group, although ineligible to receive medication through the LF MDA programme, were included as they represented a surrogate baseline indicator for the prevalence of STH in untreated children.

To calculate the number of reported and treated STH cases, health records of patients who indicated abdominal pain, passing of worms, and who were recommended to be treated with albendazole were enumerated. Other conditions that had been treated with mebendazole or albendazole were also included.

Scabies cases were defined as patients presenting with a skin condition characteristic of scabies skin disease and rashes, and who were prescribed with the standard treatment Scabex (benzyl benzoate).

### Island-level analysis

A Poisson regression model was fitted to data from each of the islands independently for both STH and scabies, with the outcome variable being the number of cases of each of the conditions. Both year and age group were considered as explanatory variables in the regression models to determine whether there was a significant decrease in recorded cases over time, and if the number of cases varied significantly between age groups. The birth-5 age group was used as the reference age group.

### District-level analysis

Scatterplots of the number of cases of the disease at the district-level each year suggested that the number of cases in some districts were intrinsically higher or lower than others. As these between-district differences cannot be fully quantified, a Poisson random effects model with a random intercept by district was fitted to the district-level case data. As with the model at the island-level, year and age group were included in the model as explanatory variables. Differences in district-level population sizes were accounted for by including the log-transformed population estimates from the 2002 national census as an offset (http://www.nbs.go.tz/takwimu/references/2002popcensus.pdf). A likelihood ratio test was undertaken in order to assess the significance of the random effects.

Ethical approval of the study was given by the Ministry of Health and Social Welfare, Zanzibar and the Ethical Committee of the Liverpool School of Tropical Medicine as part of a PhD study by KM.

## Results

### Case level analysis

#### Soil transmitted helminths

**Figure 1:**
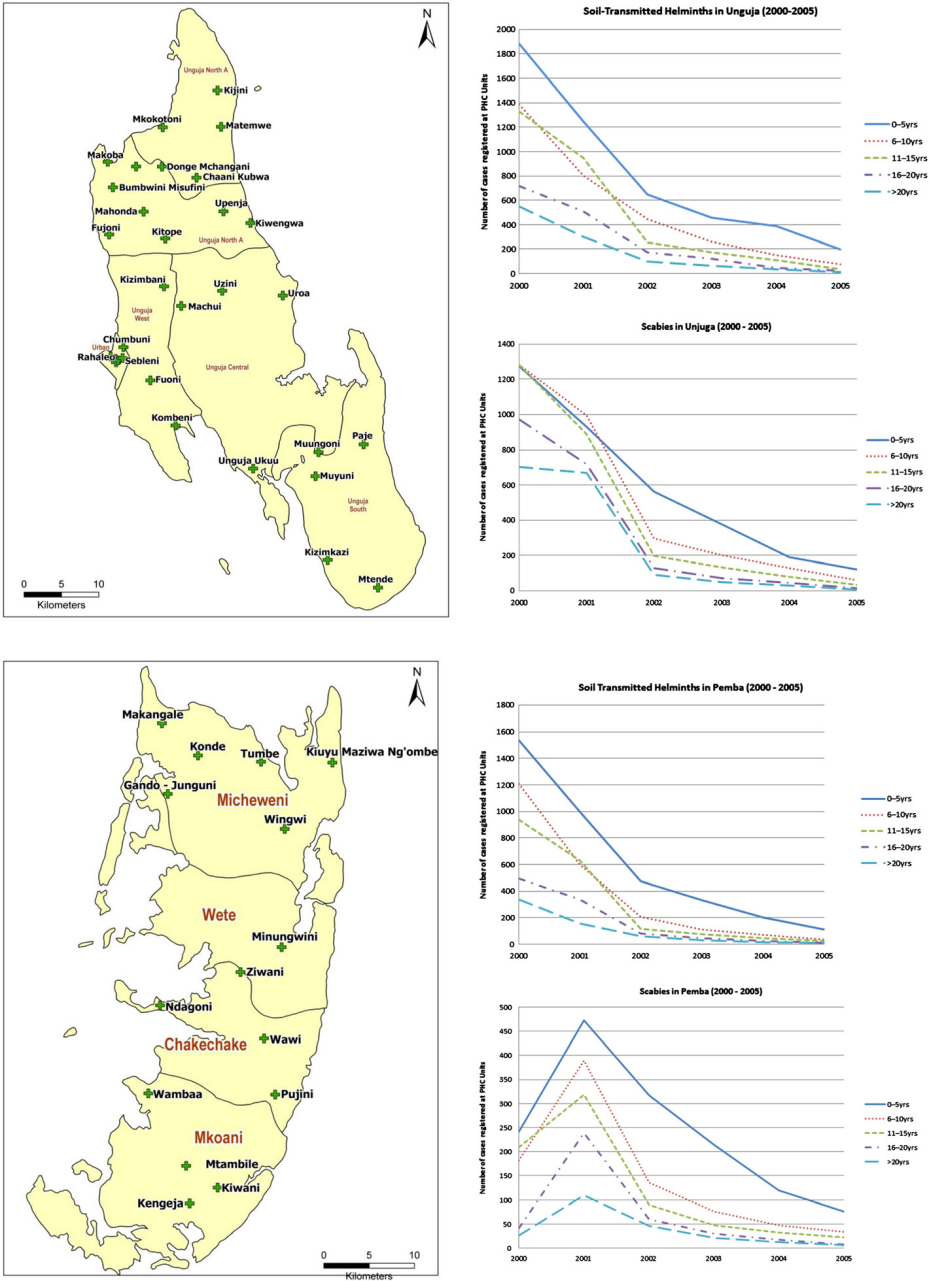
**Soil-transmitted helminths and scabies in Unguja and Pemba 2000–2005.** Number of reported cases of soil-transmitted helminths and scabies in public health units (PHC) on Unguja and Pemba, Zanzibar.

**Figure 2:**
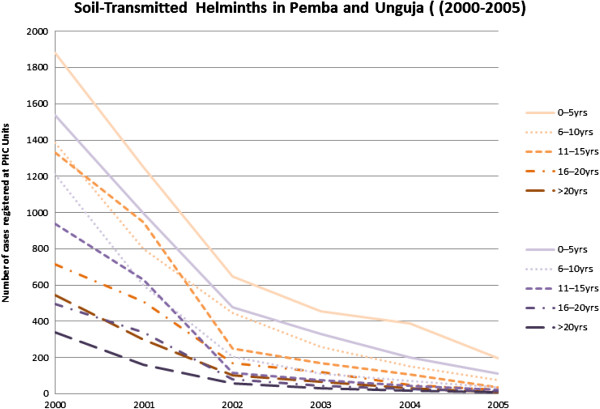
**Soil transmitted helminths in Pemba and Unguja (2000–2005).** Number of reported cases of scabies in Pemba and Unguja combined.

The results indicated that over a period of five years there was a clear decline in the number STH cases reported at health clinics on Unguja and Pemba islands in Zanzibar (Tables 1, 2 and 3). On Unguja, the largest decrease (98.17%) in reported cases was seen in the over 20 years age group. Similar trends in number of reported cases were also seen in the 11–15 year age group (97.14%), the 6–10 year group (94.44%) and the 16–20 year age group (97.06%) (Figure 1). The smallest decrease (89.63%) was in the birth-5 year age group. The highest decline was in Pemba, in the 11–15 year age group (97.44%). Other decreases in numbers of reported cases included; 16–29 years (97.38%), >20 years (97.33%), 6–10 years (96.86%). The birth-5 years age group demonstrated the lowest decline (92.97%) in number of reported cases (Figures 1 and 2).

**Table 1:** Reported cases of soil-transmitted helminths in Unguja

**Year**	**0–5 yrs**	**6–10 yrs**	**11–15 yrs**	**16–20 yrs**	**>20 yrs**
**2000**	1881	1385	1330	715	547
**2001**	1251	801	948	511	299
**2002**	650	445	252	172	101
**2003**	455	259	171	122	66
**2004**	388	153	108	49	33
**2005**	195	77	38	21	10

Legend: Cases recorded in PHCUs between 2000 and 2005.

**Table 2:** Reported cases of scabies in Unguja

**Year**	**0–5 yrs**	**6–10 yrs**	**11–15 yrs**	**16–20 yrs**	**>20 yrs**
**2000**	1273	1281	1282	973	703
**2001**	930	995	889	718	670
**2002**	563	297	197	131	90
**2003**	376	201	132	72	50
**2004**	190	129	79	47	29
**2005**	122	61	36	14	9

Legend: Cases recorded in PHCUs between 2000 and 2005.

**Table 3:** Reported cases of soil-transmitted helminths in Pemba

**Year**	**0–5 yrs**	**6–10 yrs**	**11–15 yrs**	**16–20 yrs**	**>20 yrs**
**2000**	**1539**	**1212**	**938**	**496**	**338**
**2001**	**996**	**598**	**631**	**338**	**159**
**2002**	**476**	**206**	**116**	**81**	**59**
**2003**	**332**	**113**	**78**	**46**	**33**
**2004**	**200**	**70**	**44**	**28**	**17**
**2005**	**111**	**38**	**24**	**13**	**9**

Legend: Cases recorded in PHCUs between 2000 and 2005.

**Figure 3:**
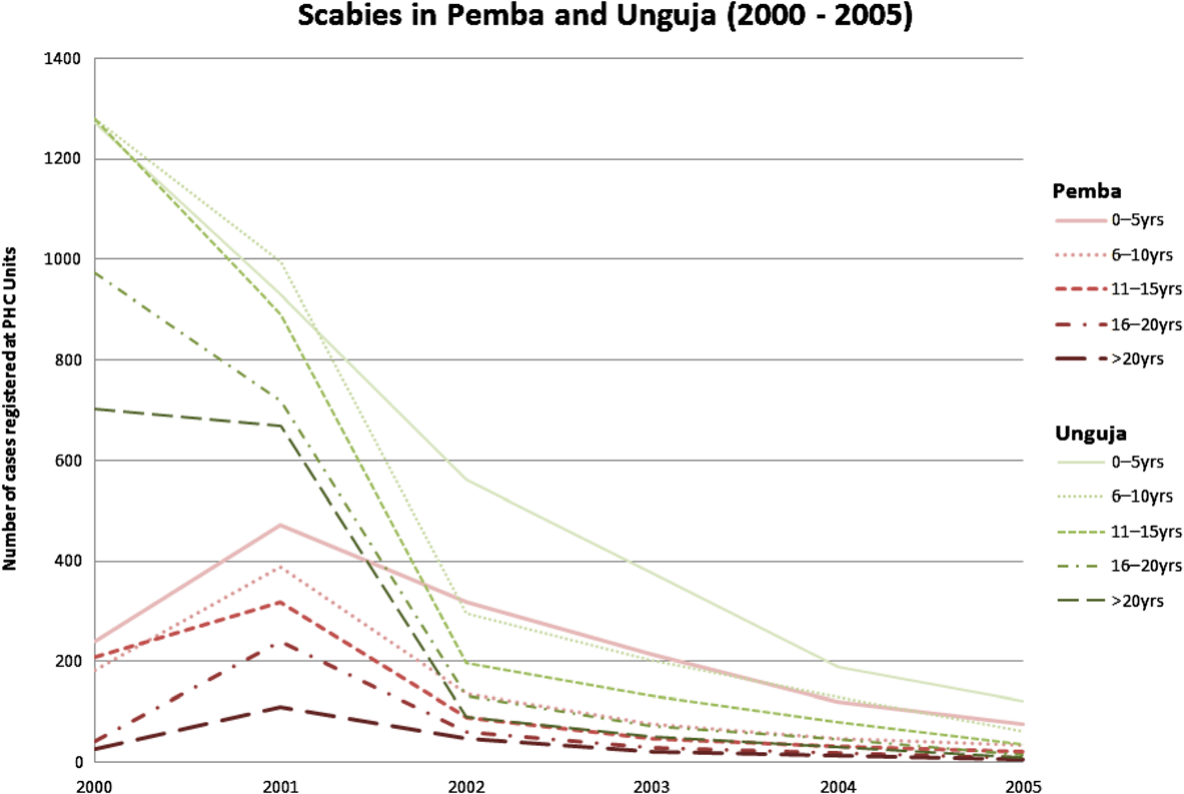
**Scabies in Pemba and Unguja (2000–2005).** Number of reported cases of soil transmitted helminths in Pemba and Unguja combined.

#### Scabies

The records of scabies declined dramatically over the period 2000–2005 with most significant decreases (98.72%) being seen on Unguja (in the over 20 year age group) (Figure 2). Other age groups on Unguja also demonstrated consistently high reductions in recorded cases; 98.56% (16–20 years), 97.19% (11–15 years), 95.24% (6–10 years) and 90.42% (birth-5 years). In Pemba, decreases in scabies cases reported were in age groups: 11–15 years (89.47%), 16–20 years (82.93%), 6–10 years (81.32%) and over 20 years (76.92%). The birth-5 years group showed a 68.46% decrease (Figures 1 and 3) and (Table 4).

**Table 4:** Reported cases of scabies in Pemba

	**0–5 yrs**	**6–10 yrs**	**11–15 yrs**	**16–20 yrs**	**>20 yrs**
**2000**	241	182	209	41	26
**2001**	472	389	318	239	109
**2002**	317	135	90	60	46
**2003**	214	76	47	30	21
**2004**	119	47	32	18	12
**2005**	76	34	22	7	6

Legend: Cases recorded in PHCUs between 2000 and 2005.

### Island-level analysis

Table 5 presents the estimated coefficients and their associated 95% confidence intervals on the log-scale for each of the four Poisson regression models fitted to the island-level data. Each of these models showed there was a statistically significant decrease in the number of cases over time. The decrease in cases over time was greater in Pemba than Unguja, whilst the effects of time appear to be greater in STH cases in comparison to scabies.

**Table 5:** Effect size and associated confidence intervals obtained from fitting a Poisson regression model to aggregated STH and scabies data from Unguja and Pemba

**Model**	**Year**		**Age 0-5**	**Age 6-10**		**Age 11-15**		**Age 16-20**		**Age > 20**	
	**Coefficient**	**95% CI**		**Coefficient**	**95% CI**	**Coefficient**	**95% CI**	**Coefficient**	**95% CI**	**Coefficient**	**95% CI**
STH Unguja	−0.5641	(−0.5769, -0.5513)	-	−0.4349	(−0.4801, -0.3900)	−0.5265	(−0.5729, -0.4803)	−1.1090	(−1.1660, -1.0526)	−1.5180	(−1.5854, -1.4522)
STH Pemba	−0.6662	(−0.6829, -0.6496)	-	−0.4907	(−0.5434, -0.4382)	−0.6910	(−0.7473, -0.6350)	−1.2938	(−1.3642, -1.2244)	−1.7820	(−1.8683, -1.6974)
Scabies Unguja	−0.3508	(−0.3720, -0.3297)	-	−0.5113	(−0.5960, -0.4272)	−0.6952	(−0.7853, -0.6061)	−1.2928	(−1.4054, -1.1826)	−1.8781	(−2.0225, -1.7386)
Scabies Pemba	−0.6388	(−0.6529, -0.6249)	-	−0.1530	(−0.2021, -0.1039)	−0.2783	(−0.3291, -0.2275)	−0.5691	(−0.6248, -0.5138)	−0.8006	(−0.8608, -0.7409)

### District-level analysis

**Figure 4:**
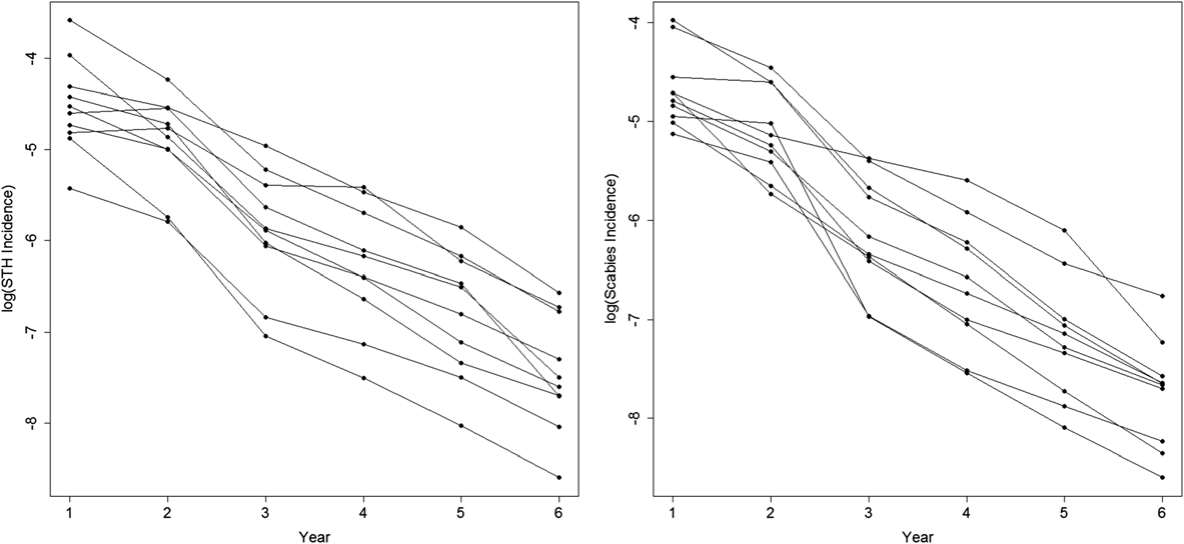
**Number of STH and scabies cases per 10,000 people on the log-scale at the district-level in Zanzibar over the period 2000–2005.** Lines connect data from the same district.

Figure 4 presents scatter plots of incidence of both STH and scabies on the log-scale at the district-level between 2000–2005. These plots show that the overall trend of cases decreases over the period of the MDA. Further, while there was a large amount of variability between the districts in a given year, the trend is broadly similar across all districts. These observations support the fitting of a Poisson random effects model with a random intercept. Table 6 presents the estimated coefficients and their associated 95% confidence intervals on the log-scale for the two Poisson random effects models fitted to the district-level data. The results obtained for the island-level models showed that there was a statistically significant decrease in the number of cases over time. In contrast to the island-level models, the effect of time was stronger in the scabies model in comparison to the STH model, although the difference was small (−0.5991 for the STH model compared to −0.6421 for the scabies model). These results also demonstrated that there was a statistically significant relationship between the number of cases and age group in both the STH and scabies models, with the risk of infection being greatest in the birth-5 year age group, following which the risk decreases with age. This relationship was more pronounced for STH. The random effects term in both of the models was significant (p<0.0001) indicating that there were between-district differences in the overall level of both diseases after accounting for differences in population sizes.

**Table 6:** Effect size and associated confidence intervals obtained from fitting a Poisson random effects model with a random intercept by district to the district-level STH and scabies data

**Model**	**Year**		**Age 0-5**	**Age 6-10**		**Age 11-15**		**Age 16-20**		**Age > 20**	
	**Coefficient**	**95% CI**		**Coefficient**	**95% CI**	**Coefficient**	**95% CI**	**Coefficient**	**95% CI**	**Coefficient**	**95% CI**
STH	−0.5991	(−0.6036, -0.5947)	-	−0.4464	(−0.4615, -0.4313)	−0.5718	(−0.5875, -0.5562)	−1.1604	(−1.1797, -1.1411)	−1.5924	(−1.6153, -1.5695)
Scabies	−0.6421	(−0.6471, -0.6371)	-	−0.1949	(−0.2121, -0.1776)	−0.3405	(−0.3585, -0.3225)	−0.6473	(−0.6671, -0.6275)	−0.9375	(−0.9594, -0.9157)

Data from Unguja and Pemba are combined.

## Discussion

A decrease in the number of STH and scabies cases diagnosed by health centre staff from 50 health facilities in Unguja and Pemba after the LF elimination programme had distributed ivermectin and albendazole between 2000–2005 was found in all age groups. We report these results conscious that the methodology we employed has limitations. However, we also consider that the method merits further investigation as MDA programmes upscale towards the milestones set by WHO with regards to achieving the maximum coverage of preventive chemotherapy by MDA for onchocerciasis, lymphatic filariasis, schistosomiasis and soil transmitted helminths (http://www.who.int/neglected_diseases/resources/en/index.html). Specifically, it is envisaged that over one billion annual treatments are required if targets for control or elimination of these diseases are to be met.

The limitations that can be identified reflect the settings of a busy health centre environment, where diagnosis is made predominantly on history and symptomatology. In the case of helminth infections, only severe or moderate cases which give rise to symptoms will present, whilst the presentation of those with low grade infection is less likely. Diagnosis of scabies is more reliable due to characteristic skin lesions presented. Despite such differences in infection status and reliability of diagnostic methods used in health centres, the trends we describe for both STH and scabies after prolonged ivermectin and albendazole distribution within the community are similar. Given the unspecific nature of clinical presentations of STH, consistency in recording STH in health centre records is important. Health staff diagnose on the basis of experience, however, as the same staff tend to be in post over many years we would argue that the data obtained can be attributed to consistency in diagnoses and prescribing. Whilst low grade and hence less intense infections would not present to clinics yet still benefit from the anthelminthic benefits of MDA, the results can be interpreted as a consistent decline in the numbers of people with moderate to severe symptoms associated with STH. Despite these limitations we believe the consistent decline in two groups of infections determined using this rapid methodology allows retrospective analysis of data. This would not be possible using classical approaches. The trends of decline were observed in two different islands, in all age groups and in 50 health centres for conditions which are biologically different in terms of transmission yet are affected by the drugs deployed in MDA, supporting the further exploration of the methodology in a more controlled setting. Importantly, the trends in scabies can be viewed as a control for the STH observations as scabies is more easily diagnosed by health staff and the scabies results mirror those of STH in both the districts and the islands. We did not record the total number of individuals who presented at these clinics during the study period.

The results are also consistent with the reported high MDA coverage [5] during the period of 2000–2005. The Global LF programme and onchocerciasis programmes are together administering MDA in around 60 countries, and over 600 million people each year are benefitting from these drugs, which have broad anthelminthic impact [3,8]. There is therefore a need to evaluate the broader synergistic impact on both STH and scabies of MDAs and the approach we describe merits consideration for the reasons we articulate below.

We suggest that the use of the gold standard approach the Kato-Katz test, as well as other more sensitive concentration techniques, would be both impractical and costly to undertake at scale as they require extensive stool collection and laboratory diagnosis. Furthermore they cannot be carried out retrospectively. Indeed, despite the extensive distribution of anthelminthics as part of LF and onchocerciasis programmes, we are not aware of any pre-MDA STH or scabies baseline data being available. While it may be appropriate to evaluate the outcomes using classical methods in limited settings, to contemplate, the use of such methods at scale when there will have been a significant reduction in the sensitivity of the test because of wide scale MDA would seem inappropriate.

National guidelines stipulate that only children of 5 years old (90 cm in height as a surrogate) and above should receive the medication for lymphatic filariasis, hence the youngest age group (birth-5 years) would not be treated. However, an observed decline in STH and scabies for this group after the initiation of MDA suggested that the treatment of the older age groups impacted on the birth-5 year old group also. This may also have been attributed to the national helminth programme, which targeted the younger age groups during the same period [8] although this confounder would not be present in the older age groups nor would influence the scabies results.

STH has been recognised as a problem in Zanzibar since surveys revealed prevalence in school aged children to be 72% for *Ascaris lumbricoides*, 94% for *Trichuris trichiura* and 95% for hookworm in Pemba [13]. This study indicated a marked decrease in reported STH diagnosis, with this reduction likely to be in those with moderate or severe infections of hookworm and *Ascaris*. Studies in Haiti and Sri Lanka indicated that significant decreases in STH were observed [14,15] following the filariasis control programme, emphasising the effectiveness of albendazole treatment.

In our study the total number of scabies cases identified was considerably lower than during the period prior to the initiation of MDA for LF. Studies by Bockarie et al. [16] showed the effectiveness of ivermectin on scabies, particularly in areas of high prevalence, where a disappearance of scabies due to the impact of ivermectin was demonstrated [16]. Scabies is diagnosed at the primary health care level when patients present with itchy papules and characteristic rash [17], although definitive techniques including dermoscopy, skin scraping and the adhesive tape test give a precise diagnosis [17]. Although mass screening and treatment of individuals affected by scabies would lead to a significant decrease in scabies prevalence, Gilmore [18] identified that it would it be difficult to sustain the implementation of such protocols over extended periods. Ivermectin is not an indicated drug for scabies despite its efficacy [7,10] and the benefits of ivermectin for scabies can only be assessed where it is used in LF and onchocerciasis programmes.

Speich et al. [19] estimated the cost of a Kato-Katz test as 1.73 US$ on Zanzibar, a figure which contrasts with the annual costs of LF MDA distribution in Tanzania of 0.26-0.54 US$ per person [20]. We consider that it is not feasible to initiate large scale stool examination using techniques which are costly, time consuming and insensitive for the assessment of the impact of MDA for LF and onchocerciasis. The approach we suggest, which despite its limitations, appears to provide consistent results, could be applied retrospectively and does not require the deployment of technical staff or the collection, transport and storage of stool samples.

## Conclusions

We propose a rapid methodology based on health records to assess the impact of MDA for lymphatic filariasis on STH and scabies. This approach would not require the extensive parasitological examination of stools, which is costly and not applicable at scale. The results suggest that distribution of albendazole and ivermectin has an impact on the number of reported cases of moderate to severe STH infections and scabies when health centre records are used as a surrogate for “gold standard” parasitological diagnosis. Further studies are required to validate the methodology to assess the impact of ivermectin and albendazole on the prevalence of STH and scabies, but we consider that the use of historic records could provide a much needed rapid assessment alternative to standard parasitological methods where sensitivity will be compromised by MDA. The method could be used retrospectively where MDA for preventive chemotherapy has already been undertaken, as well as in areas yet to upscale.

## Competing interests

The authors declare they have no competing interests.

## Authors’ contributions

KM carried out the study under the supervision of DM who initiated the idea. RD wrote the first draft of the paper adapted from the PhD thesis of KM and adapted the Figures and Tables and redrew maps. MS undertook the statistical analysis. All authors contributed to the writing of the final draft and approved the final version.
